# Lower-Limb Flexibility Profile Analysis in Youth Competitive Inline Hockey Players

**DOI:** 10.3390/ijerph17124338

**Published:** 2020-06-17

**Authors:** Antonio Cejudo, Víctor Jesús Moreno-Alcaraz, Mark De Ste Croix, Fernando Santonja-Medina, Pilar Sainz de Baranda

**Affiliations:** 1 Department of Physical Activity and Sport, Faculty of Sport Sciences, Regional Campus of International Excellence “Campus Mare Nostrum”, University of Murcia, 30100 Murcia, Spain; antonio.cejudo@um.es (A.C.); victorjm@um.es (V.J.M.-A.); psainzdebaranda@um.es (P.S.d.B.); 2 Sports and Musculoskeletal System Research Group (RAQUIS), University of Murcia, 30100 Murcia, Spain; mdestecroix@glos.ac.uk; 3 Exercise and Sport Research Centre, School of Sport and Exercise, University of Gloucestershire, Gloucester GL2 9HW, UK; 4 Department of Surgery, Pediatrics, Obstetrics and Gynecology, Faculty of Medicine, Regional Campus of International Excellence “Campus Mare Nostrum”, University of Murcia, 30100 Murcia, Spain

**Keywords:** young athlete, injury risk, athletic development, tightness, asymmetry, ROM-SPORT battery

## Abstract

During puberty, the growth of the bones is faster than that of the muscles, which may result in muscular tightness. Muscular tightness and asymmetry have been associated with an increase in injury incidence. The assessment of a joint range of motion (ROM) could help to identify athletes classified as high injury risk. The objectives of the present study were to describe the lower-extremity flexibility profile (LEFP) of youth competitive inline hockey players using the ROM-SPORT battery (I) and to identify muscular tightness and asymmetry (II). Seventy-four young players were examined for maximum passive ankle, knee, and hip ROMs. Muscle asymmetry or tightness was classified according to cutoff scores previously described. The LEFP of the 74 players was 10.8° for hip extension, 26° for hip adduction, 33.6° for ankle dorsiflexion, 38.6° for ankle dorsiflexion with knee flexed, 36.7° for hip abduction, 46° for hip internal rotation, 60.6° for hip external rotation, 65.1° for hip abduction with the hip flexed, 66.3° for hip flexion with the knee extended, 119.7° for knee flexion, and 133.7° for hip flexion. The individual analysis of the flexibility values identified tightness in all players for one or more movement, except for hip abduction. A low prevalence of asymmetries was observed (range: 5.4% to 17.6% of players) depending on the ROM.

## 1. Introduction

Inline hockey (IH) has become an increasingly popular sport in Spain during the last decade, with the senior women’s and men’s national IH teams placed in the top ten in the IH World and European Championships [1]. IH is a dynamic team-based sport characterized by constant changes in speed and direction over different distances. To achieve high levels of performance, IH players must have high levels of skating and stick handling skills such as passing, receiving, driving, hitting, or shooting [2,3,4,5]. Due to the demanding physical requirements of this sport, sports science experts consider physical fitness an essential factor to maintain optimal technical-tactical performance in competition during the season [6,7,8]. 

Muscle flexibility is one of the key components of athletic performance together with strength, endurance, speed, and coordination [6,9,10]. Several studies have demonstrated that higher levels of performance in physical-technical sports (sprint, jump, agility, shooting, dynamic balance) are related to high levels of lower-limb muscle flexibility and range of motion (ROM) [11,12,13,14,15]. In addition, it has been suggested that muscle tightness and limited ROM increase the sports injury risk [16,17,18,19].

One of the mechanisms that may contribute to muscle tightness in young athletes is the growth spurt [20]. This phenomenon consists of a rapid growth period during puberty, where the bone growth (length achieved by the extremities) is faster than the adaptation of the muscles attached to the bones [20,21]. This temporary situation (commonly known as “adolescent motor awkwardness”) contributes to a decrease in muscle–tendon extensibility in postural and biarticular muscles, which may produce substantial limitations on the range of motion (ROM) on joint extremities [22]. This mechanism explains why muscular tightness is an important factor associated with the incidence of injury in young athletes [20,21,22]. Another factor affecting the restrictions of ROM in young IH players, and therefore an increased injury risk factor, is the high requirements in terms of physical fitness of IH players [23]. The exposure of an immature musculoskeletal system to high loads (compressive, torsional, transverse, and tensile) and repeated movements used in technical actions of IH during training and competition, cause physical stress and fatigue on the muscles [24]. When such loading is not compensated with adequate management measures and enough recovery time, changes in the muscle–tendon units may result in alterations in their mechanical and neuronal properties including muscle tightness and ROM reduction [25,26]. In addition, the lack of a systematic training program for flexibility could be a significant cause in limited ROM, and therefore linked to increased injury incidence [27,28,29] and injury risk [16,30,31]. Some studies have been performed in ice hockey, which shares similar physical and technical demands with IH, and have described a relation between hip ROM values and non-contact hip injuries [32] and groin strain [33,34,35,36].

The assessment of the muscular lower-extremity flexibility profile (LEFP) of elite IH players is essential to aid physical trainers to design, adapt, control, and monitor the physical conditioning and manage the injury risk of players [37]. The LEFP is determined by eleven ROM values corresponding to the main lower limb joints movements [7,8,9,10,11,12,13,14,15,16,17,18,19,20,21,22,23,24,25,26,27,28,29,30,31,32,33,34,35,36,37]. The assessment of ROM values in these elite players allows the ability to establish reference values for young IH players [37]. Moreover, the analysis of individual flexibility profiles helps to identify players with muscular tightness and/or asymmetry in a range of lower limb ROMs [22,32]. The knowledge of ROM, specifically in young IH players, may help coaches and physical trainers improve flexibility throughout growth and maturation and establish a long-term model for athlete development [38]. 

Therefore, the objectives of the present study were to describe the LEFP of young IH players using the ROM-SPORT battery (I) and to identify players with muscular tightness and asymmetry in each movement (II). 

## 2. Method

### 2.1. Sample

Participants were selected through a convenience sample from the Technification Plan determined by the Real Federación Española de Patinaje in the 2016/17competitive season, in which the best IH players of the Regional Community were selected. Ninety competitive IH players were designated to participate in this study. Following the inclusion criteria, those who were from 8 to 15 years of age and were playing within the Real Federación Española de Patinaje categories of “Benjamin” (U11, *n* = 24) “Alevin” (U13, *n* = 30), and “Infantil” (U15, *n* = 20) were included in the study (Table 1). Due to the nature of their position, goalkeepers were not included in the current study. None of the 74 assessed participants presented a history of musculoskeletal problems in the lower limb and lower back in the last 3 months. Any players with self reported delayed onset muscle soreness on the evaluation day were excluded due to the impact this may have on players’ movement competency, joint ROM, and muscle extensibility [39,40]. Additionally, players who did not complete the descriptive questionnaire or did not complete the entire ROM-SPORT battery were excluded.

Before participation in this study, testing procedures and potential risks were fully explained to the parents and players in verbal and written form. Written informed consent was obtained from the parents of all participants.

The testing procedure was following the Declaration of Helsinki and was approved by the Ethics and Scientific Committee of the University of Murcia (Spain) [ID: 1702/2017].

### 2.2. Testing Procedure

#### 2.2.1. Questionnaire and Anthropometric Data

Before data collection, players completed a questionnaire about their IH-related background (playing position, performance level, dominant lower limb, years and months of experience in IH), and characteristics of sports participation (weekly training sessions, training hours per session, and the number of stretching exercises and duration per training session). The information obtained in the questionnaire was cross-referred with the trainer and parents to increase the objectivity. Data from the questionnaires indicated that the sample was homogeneous in potential confounding variables, such as age, body weight, stature, body mass index, training and game participation (a competitive match and 2–3 days of training per week), climatic setting, competitive level, rest periods, and sport/training experience/age. In addition, none of the players were involved in regular strength and flexibility programs during the season. Players did not regularly perform stretching exercises either in the warm-up or in the cool-down of training and competition. Data were collected 3 days into the competitive season and this time frame was selected to ensure that there was stability in the players recruited. Anthropometric measurements (body mass, stature, and body mass index) were obtained by the lead researcher at the start of the assessment session.

#### 2.2.2. The Assessment Procedure ROM

**Figure 1:**
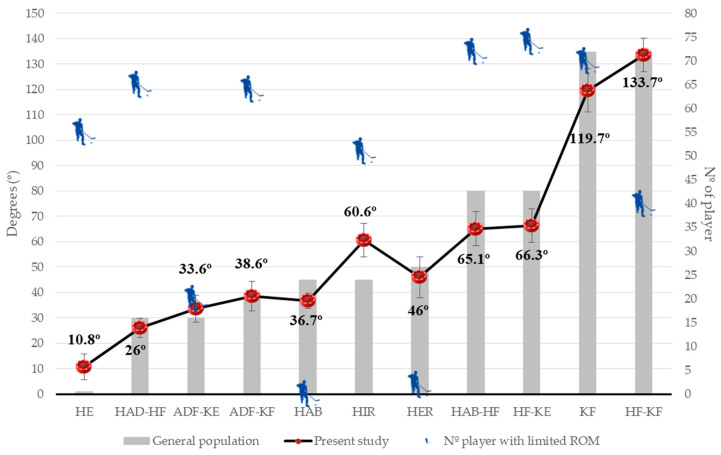
Comparison of lower-extremity flexibility profile in the 74 youth competitive inline hockey players with the general population values [37,38,39,40,51] and number of inline hockey players with limited range of motion. Hip extension test; HAD-HF: hip adduction with the hip flexed 90° extended test; ADF-KE: ankle dorsiflexion with the knee extended test; ADF-KF: ankle dorsiflexion with knee flexed test; HAB: hip abduction test; HIR, hip internal rotation test; HER: hip external rotation test; HAB-HF: hip abduction with the hip flexed 90° test; HF-KE: hip flexion with the knee extended test; KF: knee flexion test; HF-KF, hip flexion with knee flexed test.

The 11 maximum passive ROM tests of the ROM-SPORT battery were used to assess the participants [41,42]. For speed and ease of administering the battery of tests, and to minimize changing position, each participant was assessed using the methodology of the ROM-SPORT battery [7,8,9,10,11,12,13,14,15,16,17,18,19,20,21,22,23,24,25,26,27,28,29,30,31,32,33,34,35,36,37,38,39,40] (Figure 1) for the dominant and non-dominant limb in the following order: ankle dorsiflexion with knee flexed (ADF-KF) for soleus, dorsiflexion with the knee extended (ADF-KE) for gastrocnemius, hip external rotation (HER) for internal rotator muscles and hip internal rotation (HIR) external rotator muscles, hip flexion with the knee flexed (HF-KF) for gluteus maximus, hip flexion with the knee extended (HF-KE) for hamstrings, hip adduction with the hip flexed at 90° (HAD-HF) for the piriformis, hip abduction with the hip flexed at 90° (HAD-HF) for monoarticular adductors, hip abduction (HAB) for adductors, hip extension with the knee flexed (HE) for iliopsoas, and knee flexion (KF) for quadriceps of the dominant and nondominant sides were assessed following the methodology of the ROM-SPORT battery [41,42] (Figure 1). The measurement results of each of these ROMs in ascending order to determine the LEFP in the IH players of the present study [7,8,9,10,11,12,13,14,15,16,17,18,19,20,21,22,23,24,25,26,27,28,29,30,31,32,33,34,35,36,37] (Table 2).

These tests were selected because they have been considered appropriate by the American Medical Association [43] and are included in musculoskeletal measurement books [43,44,45,46,47] because they demonstrate excellent reliability [42,43,44,45,46,47,48] and validity [49,50]. In addition, the current study determined the intra-examiner reliability for each muscle flexibility measure using a test–retest design.

Before the main data collection, the absolute reliability coefficient was evaluated on 20 healthy athletes. The ROM tests were measured twice 2-weeks apart. An intraclass correlation coefficient (ICC) and the minimal detectable change at a 95% confidence interval (MDC_95_) were calculated from the subsequent measurements. Results of pre-measurement and post-measurement sessions displayed a high ICC in all the tests (0.94 to 0.97). The MDC_95_ for each ROM measure ranged from 3.7° to 6.9° [41,42]. 

Three weeks before the study, all the IH players performed a familiarization session to learn the correct technical execution of each movement. The dominant side was defined as the participant’s preferred kicking leg [37,51]. The methodology for ROM assessment was identical in both body sides, and values were obtained by the same examiners (one conducted the tests and the other ensured the proper testing position of the participants throughout the assessment maneuver) under stable environmental conditions. The ROM was measured using an ISOMED Unilevel inclinometer (Portland, Oregon) with an extendable telescopic rod. A metal goniometer with a long arm (Baseline^®^ Stainless) was used to measure the hip abduction ROM and lumbar support (Lumbosant, Murcia, Spain) was used to standardize the lumbar curvature. Before each assessment session, the inclinometer was calibrated to either 0° with the vertical or horizontal. The angle between the longitudinal axis of the mobilized segment was recorded (following its bisector) with the vertical or horizontal plane. The endpoint for each test was determined by one or both of these criteria: (1) the athlete´s feeling a strong but tolerable stretch, slightly before the occurrence of pain; (2) one or both examiners (main and assistant) detected a palpable compensatory movement that may increase the ROM [42]. The stretching sensation was held for approximately 2 s, to allow enough time to stabilize the movement and to take the measure. Two measures were recorded for each ROM test and each side. The mean of the two scores was recorded and used for statistical analysis. A 45 s rest was given between repetitions and side in each ROM test. 

At the beginning of the testing session, IH players completed a standardized warm-up consisting of 5 min jogging at moderate intensity (10–12 Borg-scale) and 15 repetitions of dynamic stretching of the evaluated muscles [52]. The warm-up session lasted about 12–15 min. Participants were examined wearing appropriate sports clothes to enable joint identification and without shoes. 

### 2.3. Statistical Analysis

The distribution of each variable was examined using the Kolmogorov–Smirnov normality test and homogeneity of variance was verified with a Levene test. Both tests confirmed that not all data was normal distribution and homoscedastic.

Descriptive statistics including mean and SDs were generated for the 11 ROM measurements. The Wilcoxon test was used to assess the relationship between the values of the dominant and non-dominant sides. The magnitude of the effect size was classified as previously described by Hopkins et al. [53] as trivial (<0.2), small (0.2 to 0.59), moderate (0.6 to 1.19), large (1.20 to 2.00), very large (2.00 to 3.99), or extremely lage (>4.0). Asymmetry was considered when the magnitude of the effect size was moderate, which is established as the minimum level of relevant effect with practical application [53], or higher than moderate. 

An individual analysis of the ROM values was performed to identify the number of players with muscle asymmetry and tightness. The asymmetry was established according to reference scores previously published in the scientific literature [37,38,39,40,41,42,43,44,45,46,47,48,49,50,51,52,53,54], which were 6° for low ROM (HE, HAD-HF, ADF-KE, ADF-KF, and HAB) and 10° for the high ROM values (HER, HIR, HAB-HF, HF-KE, KF, and HF-KF). The muscle tightness was considered when ROM values were lower than the following cut-off scores: 13° in HE [55], 30° in HAD-HF [43,44,45,46,47,48,49,50,51,52,53,54,55,56], 30° in ADF-KE, 45° in ADF-KF [44,45,46,47,48,49,50,51,52,53,54,55,56,57], 28° in HAB [55], 45° in HIR [44,45,46,47,48,49,50,51,52,53,54,55,56,57,58], 50° in HER [43], 80° in HAB-HF [59], 88° in HF-KE [60], 132° in KF [55], and 135° in HF-KF [43]. These reference cut-off values for normal or limited ROM has been associated with sport risk injury [55,60,61]. In cases where no cut-off values were previously established in the sport, we used as reference values those reported by clinical experts [44,45,46,47,56] for the general population from 18 to 60 years old. In the case of having cut-off values from both sources, the most restrictive criteria (the highest cut-off value) were used. The comparison between the mean values of the normal and muscular tightness groups in each of the ROMs assessed was calculated with the Mann–Whitney U-test. The effect size of each variable was analyzed with Pearson´s r among the groups [limited ROM vs. normal ROM] (0.0–0.39 low effect, 0.4–0.69 medium effect, and 0.7–1 high effect) [62]. 

Data were analysed using SPSS version 24 (SPSS Inc., Chicago, IL, USA). For all analyses, statistical significance was accepted at the 95% confidence level for all statistical parameters (*p* < 0.05).

## 3. Results 

Seventy-four IH players satisfied the pre-specified inclusion and exclusion criteria. 

Asymmetry (*p* ≤ 0.009) was observed in the HAD (Dom 25.4° vs. No Dom 27.3°), ADF-KF (38.2° vs. 39.1°), and HAB (37.4° vs. 36.1°); however, the effect size was categorized as trivial or small (d ≤ 0.59). Although there were statistically significant differences between the mean ROM values of the dominant and the non-dominant side for HAD, ADF-KF, and HAB, these differences are not clinically relevant (size effect: d ≤ 0.59, trivial or small) for the physical-sport practice [53]. This is why the mean values of both left and right body sides ROMs were used to describe the LEFP for youth IH players (Table 2). 

Figure 1 shows the comparison of LEFP of these IH players with the general population values. We can observe that youth IH players displayed lower values in ADF-KE (−2°), ADF-KF (−1.3°), HAB (−2.2°), HER (−9.4°), and HF-KE (−4.6°) and higher values for HE (−2.4°), HIR (−24.5°), KF (+16.7°), and HF (+2.2°) than the general population (Figure 1).

The individual analysis of each player detected asymmetry for HAB (*n* = 13), HAD-HF (*n* = 9), HE (*n* = 7), ADF-KE (*n* = 5), HER (*n* = 4), ADF-KF (*n* = 4), HIR (*n* = 2), HAB-HF (*n* = 1), HF-KE (*n* = 1), HF-KF (*n* = 1) ROMs. Concerning the muscular tightness, limited ROM was observed in the HF-KE (*n* = 74), HAB-HF (*n* = 72), KF (*n* = 70), HAD-HF (*n* = 65), ADF-KF (*n* = 64), HE (*n* = 55), HIR (*n* = 51), HF-KF (*n* = 40), ADF-KE (*n* = 20), and HER (*n* = 2) (Table 3; Figure 1). The Mann–Whitney U test displayed significant differences between the groups classified as “normal” and “limited” in the movements assessed (*p* ≤ 0.016) with a “moderate” or “high” effect size (r ≥ -0.411), except for HAB (adductors) and HF-KE (hamstrings). 

## 4. Discussion

To the best of our knowledge, this is the first study reporting the LEFP in youth IH players. The analysis of the ROM values has shown some negative sport-derived adaptations in these athletes, such as tightness and asymmetries that should be taken into account by coaches and physical trainers to design long-term training models for IH players. 

When the LEFP of youth competitive IH players was compared with the reference values of the general population (from 18 to 60 years old), a decrease in ROM was found in most hip movements (HAD-HF, HER, HAB, HAB-HF, HF-KE, and HF-KF) and for the knee flexors (KF). This decrease in ROM may be due to lower extensibility of the gluteus, pyramidal, fasciae latae tensor, hamstrings, quadriceps, and adductors among other joint tissues [63]. Most of the players included in this study fall in an age range corresponding to the maximum rate of growth [64]. This puberty process (commonly known as “adolescent motor awkwardness”) might generate a growth-related decrease in muscle flexibility (mainly in postural and biarticular muscles) that may result in significant restrictions of movements described above [21,22]. A second reason for these diminished ROM values in youth IH players may be partially explained by the impact of the systematic practice of IH (2–7 years competing in IH, 8–11 months/year, 2–3 training day/week, 3–7.5 h/week, and an IH competition a week) on the development of body posture. In addition, IH players also were not doing conditioning work that might help improve flexibility. From a biomechanical skating point of view, previous studies have shown that gluteus, pyramidal, fasciae latae tensor, hamstrings, quadriceps, and adductors are extensively used in the different phases of ice skating and speed skating [65,66,67]. A movement perpendicular to the direction of displacement (IH players kick out to the side), with a marked abduction and external rotation of the hip together with a lower plantar flexion, is characteristic of the push-off phase in skating [65,66,67]. In this phase, a total hip and knee extension is also performed [68,69,70]. The high concentric and eccentric loads of the muscles performed in skating actions could make alterations in the mechanical and neuronal properties of the muscle–tendon units, including a decrease in normal muscle extensibility and joint ROM [25,26,65,66]. 

On the contrary, IH players displayed higher HE, ADF-KE, and HIR values than the general population. It seems that the dynamic movements performed in the push-off (HE) and gliding (HIR and ADF-KE) phases increase the extensibility of the iliopsoas, external rotator muscles, and gastrocnemius [67,68]. For example, several authors have shown that optimal extensibility (iliopsoas, adductors, hamstrings, quadriceps, gluteus, and gastrocnemius), which allows a normal and specific ROM of IH players, can increase the efficiency and speed of skating, and enhance lower limb and puck handling skills [8,69,70]. 

Comparing the LEFP of youth IH players with those recently published for elite IH players [37], we observe that youth IH players displayed lower values in ADF-KE (−2°), ADF-KF (−1.3°), HAB (−2.2°), HER (−9.4°), and HF-KE (−4.6°). On the contrary, youth players showed higher values for HE (+2.4°), HIR (+24.5°), KF (+16.7°), and HF (+2.2°). Based on the ROM-SPORT battery measurement variability [42,43] which considers the minimum detectable change (MDC_95_), the only differences based on practical significance are those obtained for HIR and KF. These higher ROM in both movements is possibly the result of accumulated years of experience of the senior IH players (mean of 13.55 years of experience). 

Interestingly, our results differ from those described for college ice hockey players [32]. We observed that youth IH player displayed lower values of HE (10.8° vs. 24.3°), HAD-HF (26° vs. 27.1°), HAB (36.7° vs. 44.5°), and HF-KE (66.3° vs. 99.9°) and higher values for HIR (46° vs. 28.1°) and HER (60.6° vs. 28.9°). In addition, Tyler et al. [36] reported in professional ice hockey players higher values in ABC (45.8° vs. 36.7°) than those reported in our study. The different hip patterns, with higher HE, HAB, and HF-KE values in ice hockey players, may be due to technical movements with a greater ROM in ice hockey (i.e., trunk flexion in a defensive posture and face-offs positioning, abduction and hip extension during the forward skating stride). On the contrary, the lower HIR and HER values observed in ice hockey players may be due to a higher volume of intense loads on the hip rotator muscles that stabilize the hips, since ice hockey requires greater demands for power, speed, and hostility than those for IH. The poor flexibility shown in these joint ROMs may also reflect an adaptive response to IH practice of the articular soft-tissue that helps to improves stability at the specific joint [71].

In support of this assumption, Hogg et al. [72] found variability in ROM values between different sports. It has also been demonstrated that age (U15, U22, and senior) [4,22,54], maturation [22], and competitive level (elite, university elite, and elite U15) [73,74,75] significantly influence differences in ROM, especially in the hip. In addition, these results may be influenced by a lack of attention to flexibility training or foam rolling practices, which were limited and often non-existent based on the questionnaires completed by participants in this study. One of the main contributions of the present study is the use of the ROM-SPORT battery that allows an adequate fixation of the pelvis thanks to the help of an assistant examiner and Lumbosant, which has been shown to produce lower ROM values than those previously reported in the literature [76,77,78,79]. To compare results and to establish references values and LEFP, the protocols employed by researchers to assess ROM should be homogenized. 

Etiological studies have considered asymmetry as an important risk factor for sports injuries [80,81,82,83]. Few players (between 0.74% and 17.6% of total players depending on the type of movement) were identified as having asymmetry in the individual analysis. The number of players showing asymmetry was low, asymmetries were observed in HAB (*n* = 13), HAD-HF (*n* = 9), HE (*n* = 7), ADF-KE (*n* = 5), HER (*n* = 4), ADF-KF (*n* = 4), HIR (*n* = 2), HAB-HF (*n* = 1), HF-KE (*n* = 1), HF-KF (*n* = 1) ROMs. The results of the present study are in accordance with those reported by Cejudo et al. [37], who observed a low ratio of IH players with asymmetries (between 5% and 40% of total players). These asymmetries were reported for HIR (*n* = 8), HE (*n* = 6), HAD-HF (*n* = 6), KF (*n* = 4), HAB (*n* = 2), ADF-KE (*n* = 2), and HER (*n* = 2). A push off of both skates against the same ground conditions and the equality of turns in both directions may explain why we observed few cases of asymmetry [60,70].

Individual analysis indicated that a large number of IH players (between 2.7% and 100% of total players measured) demonstrated limited ROM in 10 of the 11 assessed movements. Cejudo et al. [37] showed similar results (between 20% and 100% of the total players measured) with limited ROM in all movements, except HAB in elite IH players. In both studies, no players showed tightness in HAB. In these studies, the cutoff score used (HAB: 28°) was selected as being the lowest cutoff score reported in the literature [55]. This value is specific to football players where the predominant hip movements are in the sagittal plane as flexion and extension, while the specific IH movement “kicks out the side” implies hip abduction [84]. The different patterns of hip movements in both sports could explain the absence of players with limited HAB. The muscular tightness observed in this study may be a result of a growth-related decrease in muscle flexibility and the high physical–technical demands of IH. In addition, another important factor that justifies the high number of players with muscle tightness is the lack of a stretching program as revealed by the questionnaires. The application of stretching will facilitate improved muscle development, whilst avoiding the negative effects (muscle tightness and asymmetry) caused by regular practice of IH [28,85,86].

The individual analysis performed in this study to identify muscle asymmetry and tightness is a useful tool to detect athletes with an increased likelihood of sports injury [22,37,87]. This individual analysis may also help to understand the mechanism of the most prevalent injuries in IH associated with muscle tightness [88] such as a sprained knee, and lumbar and adductor muscle injuries [3,5,89]. Future studies should established specific cut-off values for IH players since those available in the literature have been established for other sports, predominantly soccer. In addition, extrapolation of ROM data from ice hockey may be undertaken with a degree of caution, due to the technical differences between both hockey modalities, such as the skating phases or the type of surface [3,4,5].

### Practical Application

An important strategy for minimizing the risk of injury is to acertain the sport’s chronic adaptations to muscle flexibility during growth and maturation in youth IH players. The ROM-SPORT battery used in this study to determine the LEFP could be used by coaches and physical trainers to identify muscular tightness and asymmetry in youth IH players. This ROM assessment would be especially useful during the period of maximal rate of growth, which may result in a growth-related decrease in muscle–tendon flexibility. ROM data can be used as a reference to design stretching exercises or to increase the stretching training load to achieve optimal ROM values for this sport, and consequently, decrease the risk of injury. This stretching training should be established at a young age (6 years) to train general motor abilities including flexibility and aid movement competency, which can both enhance performance and manage injury risk [38]. 

## 5. Conclusions

The range of motion values that define the lower-extremity flexibility profile of youth IH players are 10.8° for HE, 26° for HAD-HF, 33.6° for ADF-KE, 38.6° for ADF-KF, 36.7° for HAB, 46° for HIR, 60.6° for HER, 65.1° for HAB-HF, 66.3° for HF-KE, 119.7° for KF, and 133.7° for HF-KF. The individual analysis of the lower-extremity flexibility profile identified limited ROM in all IH players for one or more of the analyzed movements, except for HAB. A low prevalence of asymmetries was observed (range between 5.4% to 17.6% of players, depending on the movement).

## Figures and Tables

**Table 1:** Demographic data (mean ± standard deviation) for the youth competitive inline hockey players in this study (*n* = 74).

Demographic Data	Minimum Value	Maximum Value	Total Value
Age (y)	8.0	15.0	11.6 ± 1.6
Body mass (kg)	27.0	71.9	49.1 ± 10.9
Stature (cm)	130.0	173.6	152.1 ± 10.3
Body mass index (kg/m^2^)	15.0	28.1	20.9 ± 3.3
Years playing IH, (y)	2.0	7.0	3.2 ± 1.5
Months per year of IH practice	8.0	11.0	9.8 ± 0.8
Days per week of IH practice	2.0	3.0	2.9 ± 0.3
Hours per week of IH practice	3.0	7.5	4.4 ± 1.3

**Table 2:** Lower-extremity flexibility profile in 74 youth competitive inline hockey players.

Range of Motion	Minimum Value	Maximum Value	Total ROM ^†^
HE (iliopsoas)	1°	23°	10.8 ± 5.0°
HAD-HF (piriformis)	16°	38°	26.0 ± 3.8°
ADF-KE (gastronemius)	23°	52°	33.6 ± 5.2°
ADF-KF (soleus)	26°	54°	38.6 ± 5.9°
HAB (adductors)	30°	45°	36.7 ± 2.9°
HIR (external rotator muscles)	27°	65°	46.0 ± 8.0°
HER (internal rotator muscles)	43°	70°	60.6 ± 6.6°
HAB-HF (monoarticulares adductors)	51°	83°	65.1 ± 6.8°
HF-KE (hamstrings)	54°	86°	66.3 ± 6.7°
KF (quadriceps)	97°	138°	119.7 ± 8.6°
HF-KF (gluteus maximus)	116°	147°	133.7 ± 6.6°

^†^ Values are expressed as mean ± standard deviation; HE: hip extension test; HAD-HF: hip adduction with hip flexed 90° extended test; ADF-KE: ankle dorsiflexion with knee extended test; ADF-KF: ankle dorsiflexion with knee flexed test; HAB: hip abduction test; HIR, hip internal rotation test; HER: hip external rotation test; HAB-HF: hip abduction with hip flexed 90° test; HF-KE: hip flexion with knee extended test; KF: knee flexion test; HF-KF, hip flexion with knee flexed test.

**Table 3:** Range of motion (ROM) classified in normal versus limited categories (mean ± standard deviation) in 74 youth inline hockey players.

Variables	Limited ROM		Normal ROM		r	*p*-Value
	*n* (%)	Mean ± SD	*n* (%)	Mean ± SD		
HE (iliopsoas)	55 (74.3%)	8.7 ± 3.5°	19 (25.7%)	17.1 ± 3.4°	−0.726	0.000
HAD-HF (piriformis)	65 (87.8%)	25.2 ± 3.1°	9 (12.2%)	32.2 ± 3.2°	−0.598	0.000
ADF-KE (gastronemius)	20 (27%)	27.7 ± 2.1°	54 (73%)	35.9 ± 4.3°	−0.696	0.000
ADF-KF (soleus)	64 (86.5%)	37.1 ± 4.7°	10 (13.5%)	48.5 ± 2.8°	−0.661	0.000
HAB (adductors)	0 (0%)	-	74 (100%)	36.7 ± 2.9°	-	-
HIR (external rotators)	51 (68.9%)	42.1 ± 6.2°	23 (31.1%)	54.7 ± 3.8°	−0.731	0.000
HER (internal rotators)	2 (2.7%)	43.0 ± 0.0°	72 (97.3%)	61.2 ± 6.1°	−0.445	0.016
HAB-HF (adductors monoarticular)	72 (97.3%)	64.6 ± 6.4°	2 (2.7%)	82.0 ± 1.4°	−0.411	0.016
HF-KE (hamstrings)	74 (100%)	66.3 ± 6.8°	0 (0%)	-	-	-
KF (quadriceps)	70 (94.6%)	118.9 ± 7.9°	4 (5.4%)	135.5 ± 2.1°	−0.440	0.001
HF-KF (gluteus maximus)	40 (54.1%)	129.0 ± 5.0°	34 (45.9%)	139.3 ± 3.1°	−0.775	0.000

Hip extension test; HAD-HF: hip adduction with hip flexed 90° extended test; ADF-KE: ankle dorsiflexion with knee extended test; ADF-KF: ankle dorsiflexion with knee flexed test; HAB: hip abduction test; HIR, hip internal rotation test; HER: hip external rotation test; HAB-HF: hip abduction with hip flexed 90° test; HF-KE: hip flexion with knee extended test; KF: knee flexion test; HF-KF, hip flexion with knee flexed test.

## References

[1] Real Federación Española de Patinaje. https://fep.es/website/index.asp?modalidad=15.

[2] Flik K., Lyman S., Marx R. (2005). American Collegiate Men’s Ice Hockey: An Analysis of Injuries. Am. J. Sports Med..

[3] Hutchinson M., Milhouse C., Gapski M. (1988). Comparison of Injury Patterns in Elite Hockey Players Using Ice versus In-Line Skates. Med. Sci. Sports Exerc..

[4] Mölsä J., Kujala U., Myllynen P., Torstila I., Airaksinen O. (2003). Injuries to the Upper Extremity in Ice Hockey: Analysis of a Series of 760 Injuries. Am. J. Sports Med..

[5] Moreno-Alcaraz V., Cejudo A., Sainz de Baranda P. (2020). Injury Types and Frequency in Spanish Inline Hockey Players. Phys. Ther. Sport.

[6] Quinney H., Dewart R., Game A., Snydmiller G., Warburton D., Bell G. (2008). A 26 Year Physiological Description of a National Hockey League Team. Appl. Physiol. Nutr. Metab..

[7] Sainz de Baranda P., Cejudo A., Ayala F., Santonja F. (2015). Perfil Óptimo de Flexibilidad Del Miembro Inferior En Jugadoras de Fútbol Sala. Rev. Int. Med. Cienc. Act. Fis. Deporte.

[8] Twist P., Rhodes T. (1993). Exercise Physiology: The Bioenergetic and Physiological Demands of Ice Hockey. Natl. Strength Cond. Assoc. J..

[9] Magnusson P., Renström P. (2006). The European College of Sports Sciences Position Statement: The Role of Stretching Exercises in Sports. Eur. J. Sport Sci..

[10] Weineck J. (2005). Entrenamiento Total.

[11] Basnett C., Hanish M., Wheeler T., Miriovsky D., Danielson E., Barr J., Grindstaff T. (2013). Ankle Dorsiflexion Range of Motion Influences Dynamic Balance in Individuals with Chronic Ankle Instability. Int. J. Sports Phys. Ther..

[12] Dill K., Begalle R., Frank B., Zinder S., Padua D. (2014). Altered Knee and Ankle Kinematics during Squatting in Those with Limited Weight-Bearing-Lunge Ankle-Dorsiflexion Range of Motion. J. Athl. Train..

[13] García-Pinillos F., Ruiz-Ariza A., Moreno del Castillo R., Latorre-Román P. (2015). Impact of Limited Hamstring Flexibility on Vertical Jump, Kicking Speed, Sprint, and Agility in Young Football Players. J. Sports Sci..

[14] Gonzalo-Skok O., Serna J., Rhea M., Marín P. (2015). Relationships between Functional Movement Tests and Performance Tests in Young Elite Male Basketball Players. Int. J. Sports Phys. Ther..

[15] Hoch M., Staton G., McKeon P. (2011). Dorsiflexion Range of Motion Significantly Influences Dynamic Balance. J. Sci. Med. Sport.

[16] Backman L., Danielson P. (2011). Low Range of Ankle Dorsiflexion Predisposes for Patellar Tendinopathy in Junior Elite Basketball Players: A 1-Year Prospective Study. Am. J. Sports Med..

[17] Tak I., Engelaar L., Gouttebarge V., Barendrecht M., Van den Heuvel S., Kerkhoffs G., Langhout R., Stubbe J., Weie A. (2017). Is Lower Hip Range of Motion a Risk Factor for Groin Pain in Athletes? A Systematic Review with Clinical Applications. Br. J. Sports Med..

[18] Witvrouw E., Danneels L., Asselman P., D’Have T., Cambier D. (2003). Muscle Flexibility as a Risk Factor for Developing Muscle Injuries in Male Professional Soccer Players: A Prospective Study. Am. J. Sports Med..

[19] Verrall G., Slavotinek J., Barnes P., Esterman A., Oakeshott R., Spriggins A. (2007). Hip Joint Range of Motion Restriction Precedes Athletic Chronic Groin Injury. J. Sci. Med. Sport.

[20] Tanner J. (1987). Issues and Advances in Adolescent Growth and Development. J. Adolesc. Health Care.

[21] Philippaerts R., Vaeyens R., Janssens M., Van Renterghem B., Matthys D., Craen R., Bourgois J., Vrijens J., Beunen G., Malina R. (2006). The Relationship between Peak Height Velocity and Physical Performance in Youth Soccer Players. J. Sports Sci..

[22] Robles-Palazón F., Ayala F., Cejudo A., De Ste Croix M., Sainz de Baranda P., Santonja F. (2020). Effects of Age and Maturation on Lower Extremity Range of Motion in Male Youth Soccer Players. J. Strength Cond. Res..

[23] Boguszewski D., Jakubowska K., Adamczyk J., Ochal A., Białoszewski D. (2017). Functional Assessment of Children Practicing Ice Hockey through Functional Movement Screen Test A-Study Design B-Data Collection C-Statistical Analysis D-Manuscript Preparation E-Funds Collection. Phys. Act. Rev..

[24] Kawałek K., Garsztka T. (2013). An Analysys of Muscle Balance in Professional Fi Eld Hockey Players. TRENDS Sport Sci..

[25] Butler D., Moseley G. (2013). Explain Pain Course Description.

[26] Fridén J., Lieber R.L. (2001). Eccentric Exercise-Induced Injuries to Contractile and Cytoskeletal Muscle Fibre Components. Acta Physiol. Scand..

[27] Opar D., Williams M., Morgan D., Shield A. (2012). Hamstring Strain Injuries: Factors That Lead to Injury and Re-Injury. Sports Med..

[28] Sato M., Mase Y., Sairyo K. (2017). Active Stretching for Lower Extremity Muscle Tightness in Pediatric Patients with Lumbar Spondylolysis. J. Med. Investig..

[29] McHugh M., Cosgrave C. (2009). To Stretch or Not to Stretch: The Role of Stretching in Injury Prevention and Performance. Scand. J. Med. Sci. Sports.

[30] Okamura S., Wada N., Tazawa M., Sohmiya M., Usuda S., Shirakura K., Shimizu T., Ibe Y. (2014). Injuries and Disorders among Young Ice Skaters: Relationship with Generalized Joint Laxity and Tightness. Open Access J. Sports Med..

[31] VandenBerg C., Crawford E., Enselman E., Robbins B., Wojtys E., Bedi A. (2017). Restricted Hip Rotation Is Correlated With an Increased Risk for Anterior Cruciate Ligament Injury. Arthrosc. J. Arthrosc. Relat. Surg..

[32] Wilcox C., Osgood C., White H., Vince R. (2015). Investigating Strength and Range of Motion of the Hip Complex in Ice Hockey. J. Sport Rehabil..

[33] Emery C., Meeuwisse W. (2001). Risk Factors for Groin Injuries in Hockey. Med. Sci. Sports Exerc..

[34] Merrifield H., Cowan R. (1973). Groin Strain Injuries in Ice Hockey:A Disparity in Muscle Strength between Both Hip Joint Adductor Muscle Groups Was Found to Be a Contributing Factor in Groin Strain Injuries. Am. J. Sports Med..

[35] Mölsä J., Airaksinen O., Näsman O., Torstila I. (1997). Ice Hockey Injuries in Finland: A Prospective Epidemiologic Study. Am. J. Sports Med..

[36] Tyler T., Nicholas S., Campbell R., Mchugh M. (2001). The Association of Hip Strength and Flexibility With the Incidence of Adductor Muscle Strains in Professional Ice Hockey Players. Am. J. Sports Med..

[37] Cejudo A., Moreno-Alcaraz V., Izzo R., Robles-Palazón F., Sainz de Baranda P. (2020). Santonja-Medina, F. Flexibility in Spanish Elite Inline Hockey Players: Profile, Sex, Tightness and Asymmetry. Int. J. Environ. Res. Public Health.

[38] Hooren B., Croix M. (2020). Sensitive Periods to Train General Motor Abilities in Children and Adolescents: Do They Exist? A Critical Appraisal. Strength Cond. J..

[39] McHugh M., Connolly D., Eston R., Gleim G. (1999). Exercise-Induced Muscle Damage and Potential Mechanisms for the Repeated Bout Effect. Sports Med..

[40] Cejudo A., Robles-Palazón F., Sainz De Baranda P. (2019). Fútbol Sala de Élite: Diferencias de Flexibilidad Según Sexo. E-Balonmano.com Rev. Ciencias del Deport..

[41] Cejudo A., Sainz de Baranda P., Ayala F., Santonja F. (2015). Test-Retest Reliability of Seven Common Clinical Tests for Assessing Lower Extremity Muscle Flexibility in Futsal and Handball Players. Phys. Ther. Sport.

[42] Cejudo A., Ayala F., Sainz de Baranda P., Santonja F. (2015). Reliability of Two Methods of Clinical Examination of the Flexibility of the Hip Adductor Muscles. Int. J. Sports Phys. Ther..

[43] Gerhardt J., Cocchiarella L., Lea R. (2002). The Practical Guide to Range of Motion Assessment.

[44] Clarkson H. (2003). Proceso Evaluativo Musculoesquelético: Amplitud Del Movimiento Articular y Test. Manual de Fuerza Muscular.

[45] Magee D. (2013). Orthopedic Physical Assessment.

[46] Norkin C., White D. (2016). Measurement Of Joint Motion: A Guide To Goniometry.

[47] Palmer M., Epler M. (2002). Fundamentos de Las Técnicas de Evaluación Musculoesquelética.

[48] Clapis P., Davis S., Davis R. (2008). Reliability of Inclinometer and Goniometric Measurements of Hip Extension Flexibility Using the Modified Thomas Test. Physiother. Theory Pract..

[49] Gogia P., Braatz J., Rose S., Norton B. (1987). Reliability and Validity of Goniometric Measurements at the Knee. Phys. Ther..

[50] Enwemeka C. (1986). Radiographic Verification of Knee Goniometry. Scand. J. Rehabil. Med..

[51] Bradley P.S., Portas M.D. (2007). The Relationship between Preseason Range of Motion and Muscle Strain Injury in Elite Soccer Players. J. Strength Cond. Res..

[52] Taylor K., Sheppard J., Hamilton L., Plummer N. (2009). Negative Effect of Static Stretching Restored When Combined with a Sport Specific Warm-up Component. J. Sci. Med. Sport.

[53] Hopkins W., Marshall S., Batterham A., Hanin J. (2009). Progressive Statistics for Studies in Sports Medicine and Exercise Science. Med. Sci. Sports Exerc..

[54] Cejudo A., Robles-Palazón F., Ayala F., De Ste Croix M., Ortega-Toro E., Santonja F., Sainz de Baranda P. (2019). Age-Related Differences in Flexibility in Soccer Players 8-19 Years Old. PeerJ.

[55] Ekstrand J., Gillquist J. (1982). The Frequency of Muscle Tightness and Injuries in Soccer Players. Am. J. Sports Med..

[56] Peterson F., Kendall E., Geise P. (2005). Kendall’s Músculos. Pruebas, Funciones y Dolor Postural.

[57] Norris C. (2007). Guía Completa de Los Estiramientos.

[58] Alter M. (2004). Los Estiramientos.

[59] Surgeons American Academy of Orthopaedic (1965). Joint Motion: Method of Measuring and Recording.

[60] Witvrouw E., Bellemans J., Lysens R., Danneels L., Cambier D. (2001). Intrinsic Risk Factors for the Development of Patellar Tendinitis in an Athletic Population. Am. J. Sports Med..

[61] Malliaras P., Cook J., Kent P. (2006). Reduced Ankle Dorsiflexion Range May Increase the Risk of Patellar Tendon Injury among Volleyball Players. J. Sci. Med. Sport.

[62] Pallant J. (2007). SPSS Survival Manual: A Step by Step Guide to Data Analysis Using SPSS.

[63] Kapandji A. (2007). Fisiología Articular T2: Miembro Inferior.

[64] Mirwald R.L., Baxter-Jones G., Bailey D., Beunen G. (2002). An Assessment of Maturity from Anthropometric Measurements Saskatchewan Growth and Development Studyn View Project Pediatric Bone Mineral Accrual Study View Project. Med. Sci. Sports Exerc..

[65] Marino G. (1983). Selected Mechanical Factors Associated with Acceleration in Ice Skating. Res. Q. Exerc. Sport.

[66] Pearsall D., Turcotte R., Murphy S. (2000). Biomechanics of Ice Hockey. Exerc. Sport Sci..

[67] Shell J., Robbins S., Dixon P., Renaud P., Turcotte R., Wu T., Pearsall D. (2017). Skating Start Propulsion: Three-Dimensional Kinematic Analysis of Elite Male and Female Ice Hockey Players. Sports Biomech..

[68] De Koning J., Thomas R., Berger M., De Groot G., Van Ingen Schenau G. (1995). The Start in Speed Skating: From Running to Gliding. Med. Sci. Sports Exerc..

[69] De Koning J., De Groot G. (1991). Coordination of Leg Muscles during Speed Skating. J. Biomech..

[70] Minkoff J., Varlotta G., Simonson B. (1994). Ice Hockey.

[71] Ostojic S., Stojanovic M. (2007). Range of Motion in the Lower Extremity: Elite vs Non-Elite Soccer Players. Serb. J. Sports Sci..

[72] Hogg J., Schmitz R., Nguyen A., Shultz S. (2018). Lumbo-Pelvic-Hip Complex Passive Hip Range-of-Motion Values Across Sex and Sport. J. Athl. Train..

[73] Battista R.A., Pivarnik J.M., Dummer G.M., Sauer N., Malina R.M. (2007). Comparisons of Physical Characteristics and Performances among Female Collegiate Rowers. J. Sports Sci..

[74] Donaldson L., Li B., Cusimano M. (2014). Economic Burden of Time Lost Due to Injury in NHL Hockey Players. Inj. Prev..

[75] De la Fuente A., Gómez-Landero L. (2019). Motor Differences in Cadet Taekwondo Athletes According to Competition Level. Rev. Int. Med. y Ciencias la Act. Fis. y del Deport..

[76] Zawadka M., Skublewska-Paszkowska M., Gawda P., Lukasik E., Smolka J., Jablonski M. (2018). What Factors Can Affect Lumbopelvic Flexion-Extension Motion in the Sagittal Plane?: A Literature Review. Hum. Mov. Sci..

[77] Gajdosik R., Simpson R., Smith R., Dontigny R. (1983). Pelvic Tilt Intratester Reliability of Measuring the Standing Position and Range of Motion. Mont. Chapter Am. Phys. Ther. Assoc..

[78] Bohannon R., Gajdosik R., LeVeau B. (1985). Relationship of Pelvic and Thigh Motions During Unilateral and Bilateral Hip Flexion. Phys. Ther..

[79] Santonja-Medina F., Santonja-Renedo S., Cejudo A., Ayala F., Ferrer V., Pastor A., Collazo-Diéguez M., Rodríguez-Ferrán O., Andújar P., Sainz de Baranda P. (2020). Straight Leg Raise Test: Influence of Lumbosant© and Assistant Examiner in Hip, Pelvis Tilt and Lumbar Lordosis. Symmetry.

[80] Arnason A., Sigurdsson S., Gudmundsson A., Holme I., Engebretsen L., Bahr R. (2004). Risk Factors for Injuries in Football. Am. J. Sports Med..

[81] Devan M., Pescatello L., Faghri P., Anderson J. (2004). A Prospective Study of Overuse Knee Injuries among Female Athletes with Muscle Imbalances and Structural Abnormalities. J. Athl. Train..

[82] Fousekis K., Tsepis E., Poulmedis P., Athanasopoulos S., Vagenas G. (2011). Intrinsic Risk Factors of Non-Contact Quadriceps and Hamstring Strains in Soccer: A Prospective Study of 100 Professional Players. Br. J. Sports Med..

[83] Hrysomallis C. (2009). Hip Adductors’ Strength, Flexibility, and Injury Risk. J. Strength Cond. Res..

[84] Budarick A., Shell J., Robbins S., Wu T., Renaud P., Pearsall D. (2018). Ice Hockey Skating Sprints: Run to Glide Mechanics of High Calibre Male and Female Athletes. Sports Biomech..

[85] Rubini E., Costa A., Gomes P. (2007). The Effects of Stretching on Strength Performance. Sports Med..

[86] Iwata M., Yamamoto A., Matsuo S., Hatano G., Miyazaki M., Fukaya T., Fujiwara M., Asai Y., Suzuki S. (2019). Dynamic Stretching Has Sustained Effects on Range of Motion and Passive Stiffness of the Hamstring Muscles. J. Sci. Med. Sport.

[87] López-Valenciano A., Ayala F., Vera-García F., De Ste Croix M., Hernández-Sánchez S., Ruiz-Pérez I., Cejudo A., Santonja F. (2019). Comprehensive Profile of Hip, Knee and Ankle Ranges of Motion in Professional Football Players. J. Sports Med. Phys. Fitness.

[88] Stuart M., Smith A. (1995). Injuries in Junior A Ice Hockey: A Three-Year Prospective Study. Am. J. Sports Med..

[89] Varlotta G., Lager S., Nicholas S., Browne M., Schlifstein T. (2000). Professional Roller Hockey Injuries. Clin. J. Sport Med..

